# Items of Local Interest

**Published:** 1914-07

**Authors:** 


### Items of Local Interest.

KLEBERG COUNTY is erecting a modern hospital at Kingsville. The building will be one of the best equipped county hospitals in the State and will cost $50,000. It will be of reinforced concrete.

DR. CROSTHWAIT, of Waco, has promised a paper on "The Advancement in Medical Education in Texas." We hope to be able to give our readers this interesting contribution in the August issue of the "Red Back."

SUTHERLAND SPRINGS is to have a new sanitarium, on which work has already begun. It is located near the famous pavilion at that popular resort, and has a beautiful stretch of park in front of it. It will cost about $10,000, and will be modern in every respect.

TEMPLE has awakened to the importance of a clean-up crusade, and has sent for Dr. M. M. Carrick, the famous Texas sanitary specialist, to get his advice. The city council will direct the movement which will be under the management of a commission composed of Dr. J. M. Murphy, W. A. Brady and Alderman W. F. Blum.

DR. E. M. MOSELY, T. H. Cobble and J. F. Johnson, of Rusk, have recently moved into a suite of four rooms that have been especially fitted up for them with electric lights and fans, bath room, running water, and all the modern fixtures. They have a large, cool reception room, and all the rooms have southern exposures.

DR. E. M. WOOD APPOINTED ON STATE BOARD OF MEDICAL EXAMINERS.—Dr. E. M. Wood, Secretary of the Williamson County Medical Society, was appointed by Governor Colquitt on the State Board of Medical Examiners, to succeed Dr. J. H. Evans, deceased. Dr. Wood is well qualified for the position. The appointment was made May 21st.—*Houston Post.*

THE editor of the JOURNAL had the pleasure of a visit to Dr. F. C. Floeckinger's sanitarium at Taylor, while attending a meeting of the Williamson County Medical Association, and was surprised to find such a well equipped hospital. Dr. Floeckinger is an enthusiastic surgeon who stands well in advance in the profession and who mixes study and brains with his surgery.

---

MARY E. HAAG, visiting nurse for the Houston Settlement House, has made her first report, which is quite interesting. It includes the period from June 8th to 30th. The total number of treatments at the dispensaries were 168. Visits made by the nurse, including nursing, instruction and investigation, totaled 139. Mothers were advised in twenty-five cases, and seven visits were made to expectant mothers. Drs. M. B. Stokes, C. W. Hoeflich, J. B. Burditt and J. C. Michael gave their services to the Settlement House, and others have tendered their services during the summer.

---

DR. JOE GILBERT, of Austin, has sailed for London, where he will attend the Congress of Surgeons, spending two months abroad in study. While abroad, Dr. Gilbert will represent the TEXAS MEDICAL JOURNAL, sending letters that will interest its readers and dealing with medical and surgical topics. He bears letters of introduction from a number of prominent American firms to leading medical journals and physicians and surgeons in Europe, and has been commissioned by the "Red Back" to secure for it the best papers presented before the Congress of Surgeons, these papers to appear in future issues.

---

MEDICAL EXAMINATION.—The State Board of Medical Examiners, which is composed of the following physicians, Dr. J. F. Bailey, President, Waco; Dr. W. L. Crosthwait, Secretary, Waco; Dr. M. E. Daniel, Chairman of the Executive and Reciprocity Committee, Honey Grove; Dr. T. J. Crowe, Chairman of the College Committee, Dallas; Dr. G. L. Baber, Winnsboro; Dr. E. L. Osborne, Cleburne; Dr. S. L. Scothorne, Dallas; Dr. H. C. Morrow, Austin; Dr. G. W. Johnson, San Antonio; Dr. E. M. Wood, Georgetown, met in Austin, June 25th. There were about one hundred and fifty applicants present to take the examination. Of this number, twelve were Mexicans and nine were negroes. In the next issue of the JOURNAL we hope to be able to give a complete list of all those who passed and to whom diplomas will be issued.

---

EXAMINATION REQUIREMENTS.—At the last meeting of the Board of Medical Examiners the following interesting announcement was made: Beginning with the coming college year the examination board will require of students that they secure a permit from the board, based upon an application, showing they have 14 units in a standard affiliated high school, seminary or academy and an additional college year consisting of 5 college units; one each in biology, chemistry, physics and a modern language; the other to be elective. With the additional provision that they may be conditioned on the college year or a part thereof until the beginning of their junior year in medicine. In other words, students may matriculate with 14 accepted units and sufficient credits on their college year as will enable them to complete their full college year before the beginning of their junior year.

---

THE Williamson County Medical Association met at Taylor on June 8th. While there were a number of physicians present from different parts of the county, the attendance, owing to the excessive heat, was small. Addresses were made by Dr. Z. T. Scott, Dr. C. E. Gilliland and Mrs. F. E. Daniel, of Austin, and those were discussed by the other physicians present. Among those present were Dr. E. M. Thomas, of Georgetown, presiding; Dr. E. M. Wood, of Georgetown, Secretary; Drs. Edmond Doak, J. I. Collier, R. B. Bledsoe, F. C. Floeckinger, W. G. Jones, J. C. Thomas, of Taylor; Dr. W. L. Helm, of Jonah; Dr. O. T. Bundy, of Hutto. Dr. Doak met the visitors from Austin at the train and took them to lunch, and Mrs. Doak gave her presence to the meeting. Refreshing cool drinks were served during the session, and the Taylor physicians did all they could to make the meeting pleasant. The papers read by Drs. Scott and Gilliland were especially interesting and helpful. Dr. Scott's paper was an able treatise on "The Ductless Glands. Dr. Gilliland has promised his paper for a future issue, his theme being "The Value of the X-Ray in Diagnosis."

---

CONTRIBUTING EDITORS.—The TEXAS MEDICAL JOURNAL feels somewhat proud of its list of contributing editors for the coming year, which includes many of the most eminent men in the profession in the United States. The following is a partial list, to which addition will be made from time to time, all of them being so well known as to need no introduction to the medical world: Dr. H. Sheridan Baketel, New York, editor *Medical Times;* Dr. M. H. Boerner, Austin, Texas; Dr. G. Henri Bogart, Paris, Ill.; Dr. M. M. Carrick, Dallas, Texas; Dr. Edward H Carey, Dean Medical Department, Baylor University, Dallas,

Texas; Dr. W. L. Crosthwaite, Waco, Texas; Dr. H. W. Cummings, Hearne, Texas; Dr. Oscar Dowling, New Orleans, President Louisiana State Board of Health; Dr. May Agnes Hopkins, Dallas, Texas; Dr. A. W. Fly, Galveston, Texas; Dr. G. B. Foscue, Waco, Texas; Dr. M. B. Grace, Seguin, Texas; Dr. Joe Gilbert, Austin, Texas; Dr. Charles W. Hughes, St. Louis, editor *Alienist and Neurologist;* Dr. James G. Kiernan, Chicago; Dr. George H. Lee, Galveston, Texas; Dr. Frank Paschal, San Antonio; Dr. John Preston, Austin, Texas, Superintendent State Lunatic Asylum; Dr. Bacon Saunders, Fort Worth, Texas; Dr. Allen J. Smith, Philadelphia, Medical Department, University of Pennsylvania; Dr. Ralph Steiner, Austin, Texas, President Texas State Board of Health; Dr. John S. Turner, Dallas, Texas; Dr. Charles Venable, San Antonio, Texas; Dr. E. S. Goodhue, Holualoa, Kona, Hawaii.

